# Temozolomide combined with irinotecan regresses a cisplatinum-resistant relapsed osteosarcoma in a patient-derived orthotopic xenograft (PDOX) precision-oncology mouse model

**DOI:** 10.18632/oncotarget.22892

**Published:** 2017-12-04

**Authors:** Kentaro Igarashi, Kei Kawaguchi, Tasuku Kiyuna, Kentaro Miyake, Masuyo Miyake, Yunfeng Li, Scott D. Nelson, Sarah M. Dry, Arun S. Singh, Irmina A. Elliott, Tara A. Russell, Mark A. Eckardt, Norio Yamamoto, Katsuhiro Hayashi, Hiroaki Kimura, Shinji Miwa, Hiroyuki Tsuchiya, Fritz C. Eilber, Robert M. Hoffman

**Affiliations:** ^1^ AntiCancer, Inc., San Diego, California, USA; ^2^ Department of Surgery, University of California, San Diego, California, USA; ^3^ Department of Orthopaedic Surgery, Kanazawa University, Kanazawa, Japan; ^4^ Department of Pathology, University of California, Los Angeles, California, USA; ^5^ Division of Hematology-Oncology, University of California, Los Angeles, California, USA; ^6^ Division of Surgical Oncology, University of California, Los Angeles, California, USA; ^7^ Department of Surgery, Yale School of Medicine, New Haven, Connecticut, USA

**Keywords:** osteosarcoma, PDOX, nude mice, temozolomide, irinotecan

## Abstract

Relapsed osteosarcoma is a recalcitrant tumor. A patient's cisplatinum (CDDP)-resistant relapsed osteosarcoma lung metastasis was previously established orthotopically in the distal femur of mice to establish a patient-derived orthotopic xenograft (PDOX) model. In the present study, the PDOX models were randomized into the following groups when tumor volume reached 100 mm^3^: G1, control without treatment; G2, CDDP (6 mg/kg, intraperitoneal (i.p.) injection, weekly, for 2 weeks); gemcitabine (GEM) (100 mg/kg, i.p., weekly, for 2 weeks) combined with docetaxel (DOC) (20 mg/kg, i.p., once); temozolomide (TEM) (25 mg/kg, p.o., daily, for 2 weeks) combined with irinotecan (IRN) (4 mg/kg i.p., daily for 2 weeks). Tumor size and body weight were measured with calipers and a digital balance twice a week. After 2 weeks, all treatments significantly inhibited tumor growth except CDDP compared to the untreated control: CDDP: *p* = 0.093; GEM+DOC: *p* = 0.0002, TEM+IRN: *p* < 0.0001. TEM combined with IRN was significantly more effective than either CDDP (*p* = 0.0001) or GEM combined with DOC (*p* = 0.0003) and significantly regressed the tumor volume compared to day 0 (*p* = 0.003). Thus the PDOX model precisely identified the combination of TEM-IRN that could regress the CDDP-resistant relapsed metastatic osteosarcoma PDOX.

## INTRODUCTION

Osteosarcoma is a recalcitrant tumor with greatest is incidence in adolescence and in the seventh and eighth decades. First-line therapy for osteosarcoma is high-dose methotrexate (MTX), cisplatinum (CDDP), doxorubicin (DOX), and ifosfamide, which is ineffective in metastatic osteosarcoma with less than 20% long-term survival and has not improved for many years [1–8].

Temozolomide (TEM) has been tested pre-clinically against osteosarcoma cells combined with a molecular targeting drug [9] and as a single-agent against osteosarcoma xenograft models [10] as well as in combination with irinotecan (IRN) in a patient-derived orthotopic xenograft (PDOX) model or rhabdomysarcoma [11].

Trabectedin (TRAB) is an alkylating agent derived from the Caribbean tunicate, Ecteinascidia turbinate [11] which has been tested on liposarcoma and leiomyosarcoma patients [12, 13], TRAB arrests cells in the G_2_/M phase of the cell cycle [14], TRAB has shown efficacy against CDDP-resistant bone cancer *in vitro* [15]. TRAB is marketed as Yondelis (Johnson & Johnson, Raritan, NJ) for leiomyosarcoma and liposarcoma [16]. TRAB has been used for recurrent osteosarcoma patients with a 12% partial response rate [17].

The combination of gemcitabine (GEM) and docetaxel (DOC) may be synergistic due to their complementary mechanisms of action of arresting cells in different phases of the cell cycle [19].

We previously reported that a subcutaneous transplant nude-mouse model of a CDDP-resistant ostoeosarcoma lung metastasis was regressed by tumor-targeting *Salmonella typhimurium* A1-R (*S. typhimurium* A1-R), but was only partially sensitive to the molecular-targeting drug sorafenib, which did not arrest its growth. *S. typhimurium* A1-R was significantly more effective than sorafenib [18].

Toward the goal of individualized precision oncology, our laboratory pioneered the PDOX nude mouse model with the technique of surgical orthotopic implantation (SOI), including pancreatic [19–22], breast [23], ovarian [24], lung [25], cervical [26], colon [27–29], and stomach cancer [30], sarcoma [31–35] and melanoma [36–38].

We subsequently developed a PDOX model from the CDDP-resistant osteosarcoma lung metastasis that recurred after adjuvant CDDP treatment of the patient [8]. The CDDP-resistant metastatic osteosarcoma PDOX was sensitive to TEM and TRAB, but not CDDP. These results showed that the PDOX model of the CDDP-resistant osteosarcoma lung-metastasis could identify potentially, highly-effective drugs for this recalcitrant disease, while accurately maintaining the CDDP resistance of the tumor in the patient [8].

In the present study, we evaluated the efficacy TEM combined with IRT, compared to GEM combined with DOC compared to CDDP on the PDOX model of CDDP-resistant relapsed osteosarcoma.

## RESULTS AND DISCUSSION

### Efficacy of CDDP alone, GEM combined with DOC and TEM combined with IRN on the CDDP-resistant metastatic osteosarcoma PDOX mouse model

**Figure 1:**
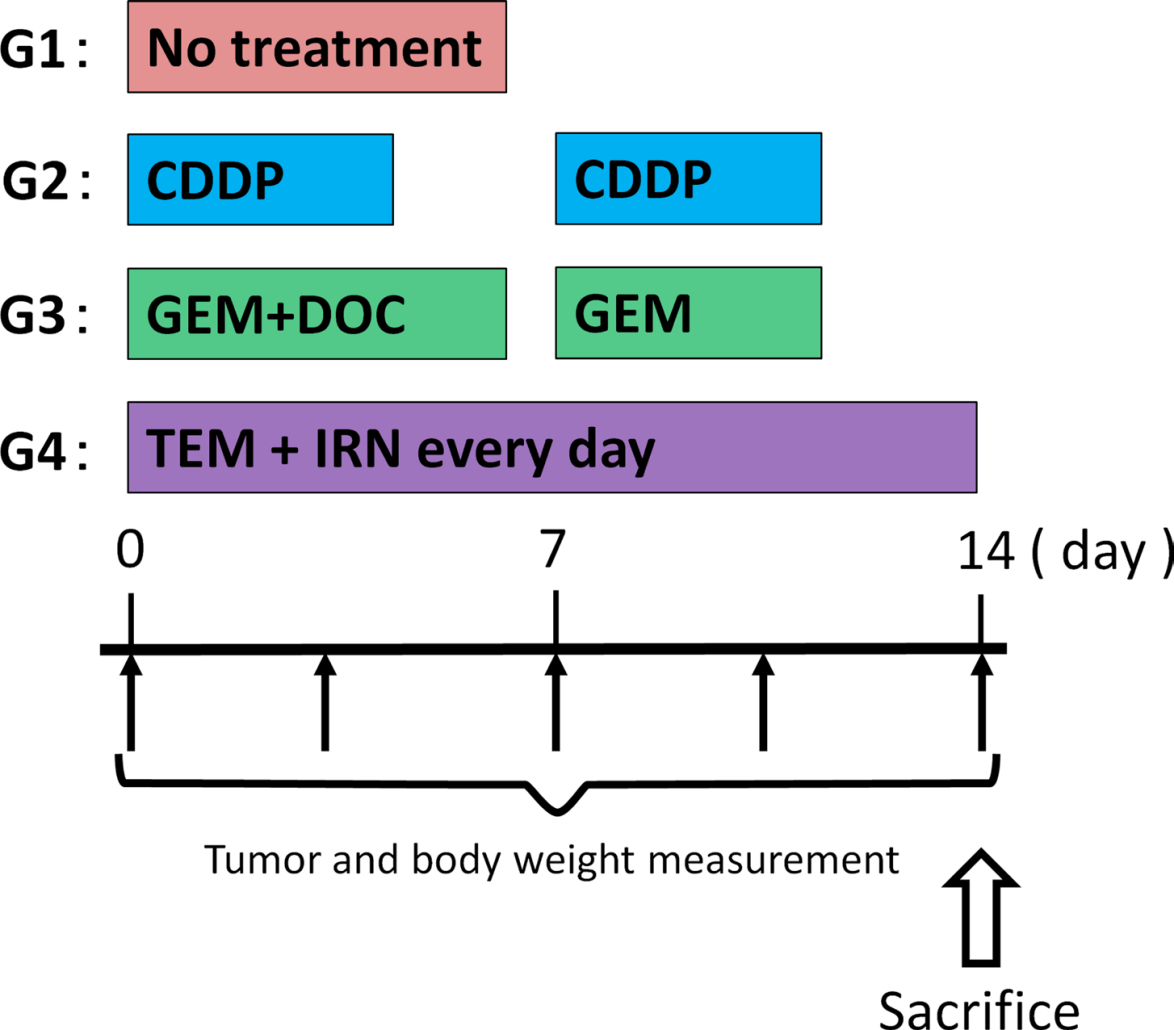
Treatment schema

**Figure 2:**
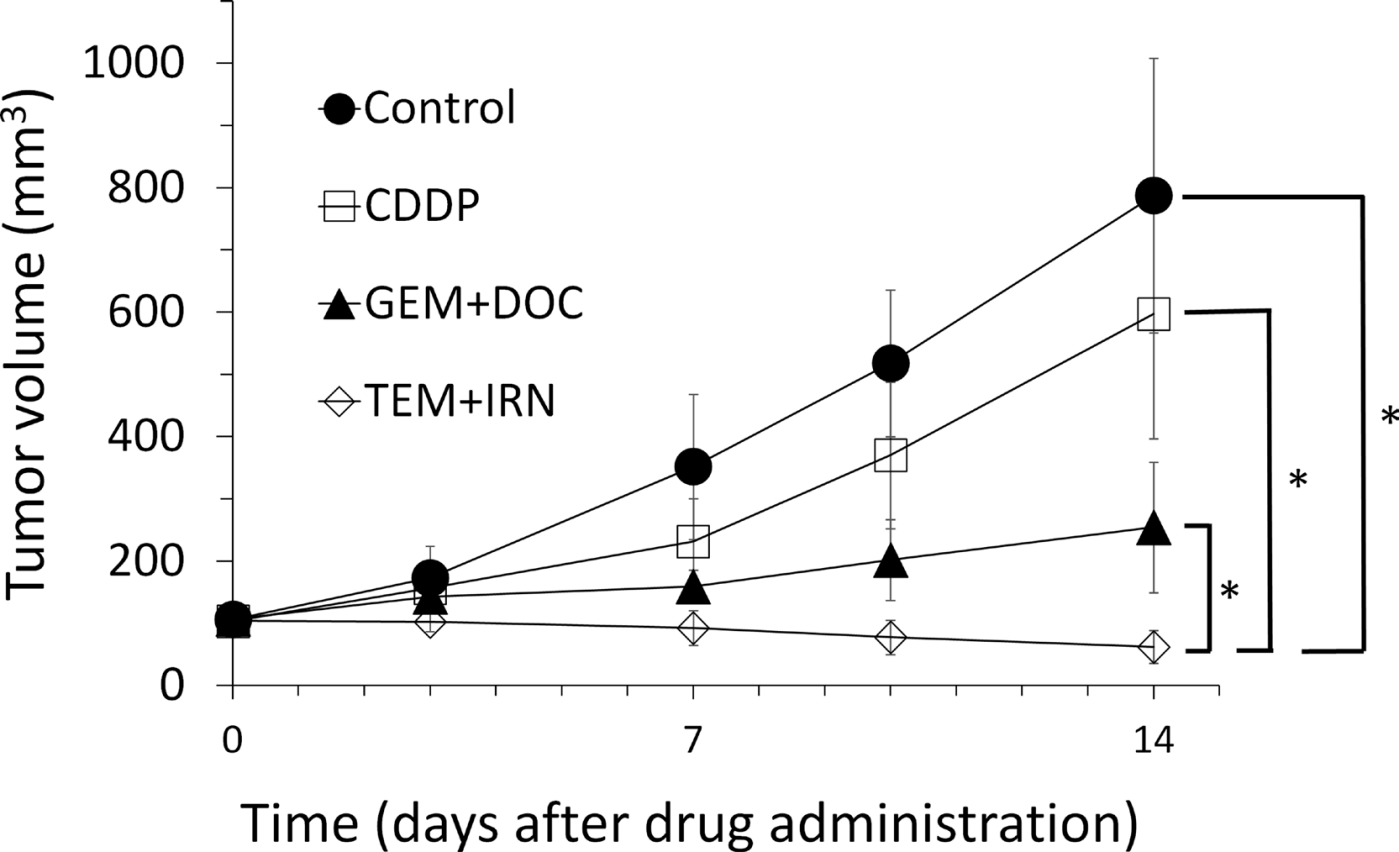
Quantitative efficacy of treatment Tumor volume was measured at the indicated time points after the onset of treatment. Please see the Materials and Methods for details. *N* = 8 mice/group. ^*^p < 0.001

**Figure 3:**
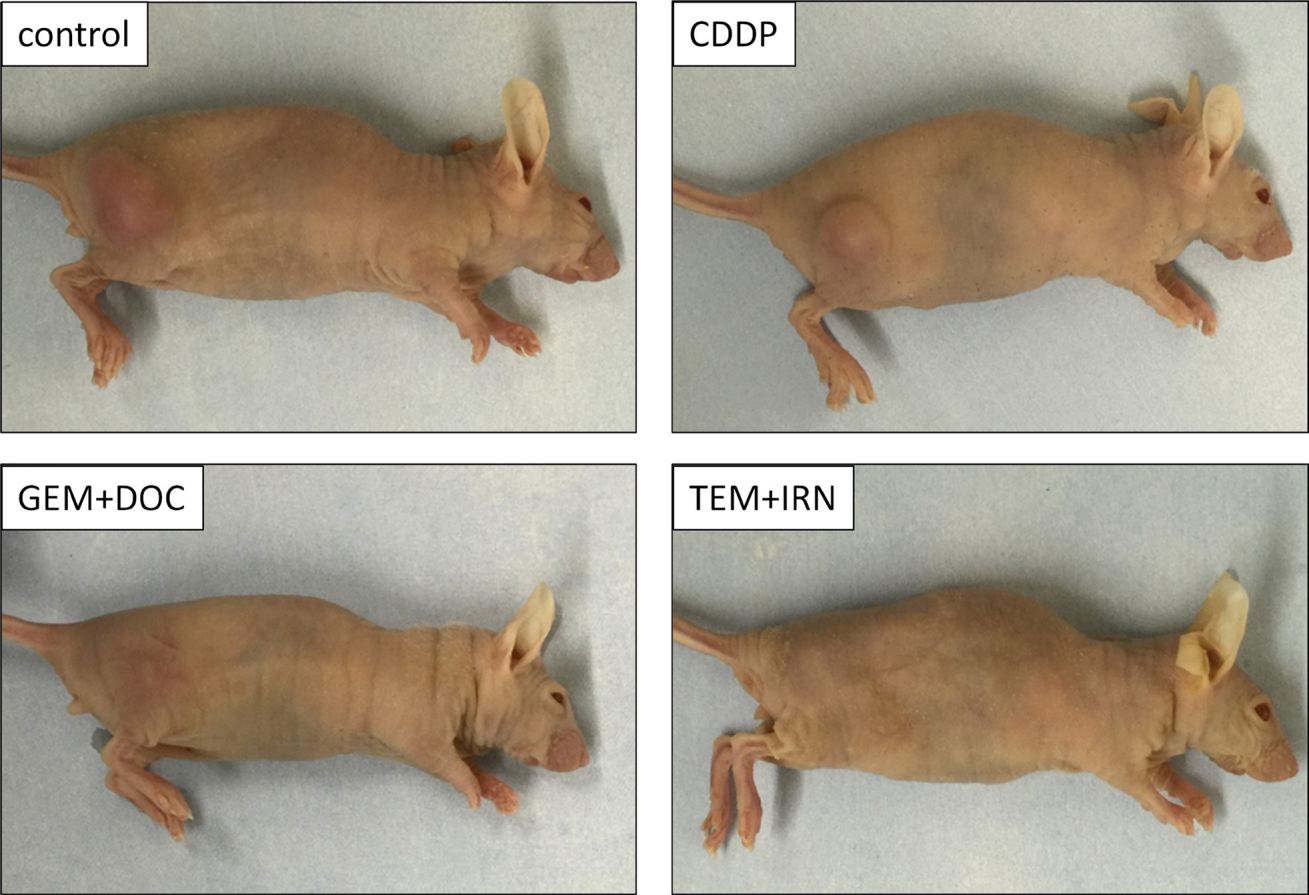
Photographs of treated and untreated tumors Photos of representative treated and untreated osteosarcoma PDOX models.

After 2 weeks, all treatments, except CDDP, significantly inhibited tumor growth compared to untreated control: CDDP: *p* = 0.093; GEM+DOC: *p* = 0.0002, TEM+IRN: *p* < 0.0001. TEM combined with IRN was significantly more effective than either CDDP (*p* = 0.0001) or GEM combined with DOC (*p* = 0.0003) and significantly regressed the tumor volume compare to day 0 (*p* = 0.003) (Figures 1–3).

The CDDP-resistant metastatic osteosarcoma PDOX faithfully replicated the CDDP-resistance of the tumor in the patient. The PDOX model could also identify the TEM-IRN combination which could regress the tumor indicating potential for efficacy in the patient [39]. The utility of the PDOX model is to match the drug to the patient. The TEM+IRN combination appears most promising as TEM alone did not regress the tumor [8].

### Effect of treatment on the body weight of PDOX models

**Figure 4:**
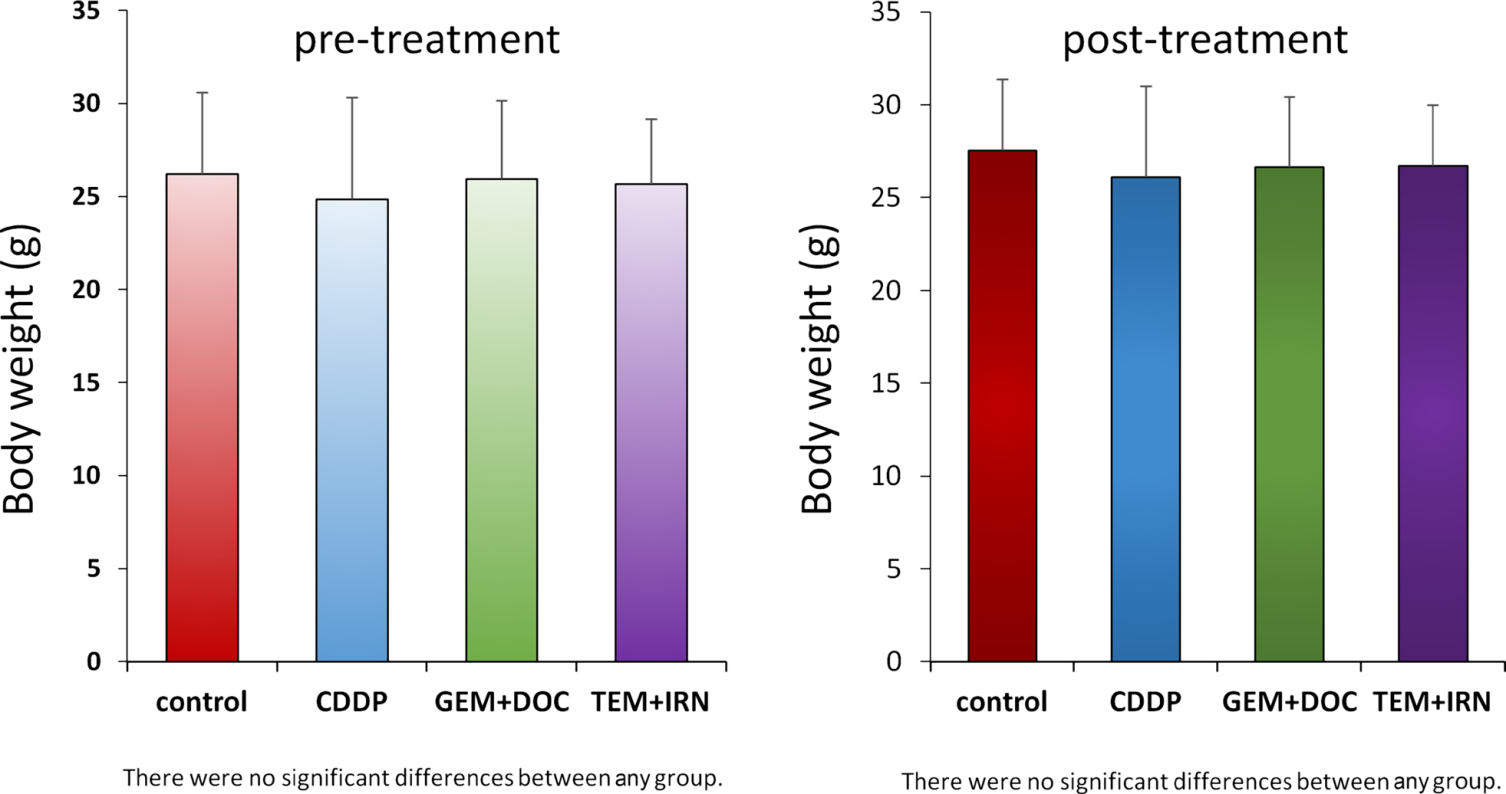
Effect of treatments on mouse body weight Bar graph shows body weight in each group at pre-treatment and 2 weeks after drug administration. There were no significant differences between each group.

The body weight of treated mice was not significantly different in any group (Figure 4). There were no animal deaths in any group.

### Effect of treatment on the histology of PDOX models

**Figure 5:**
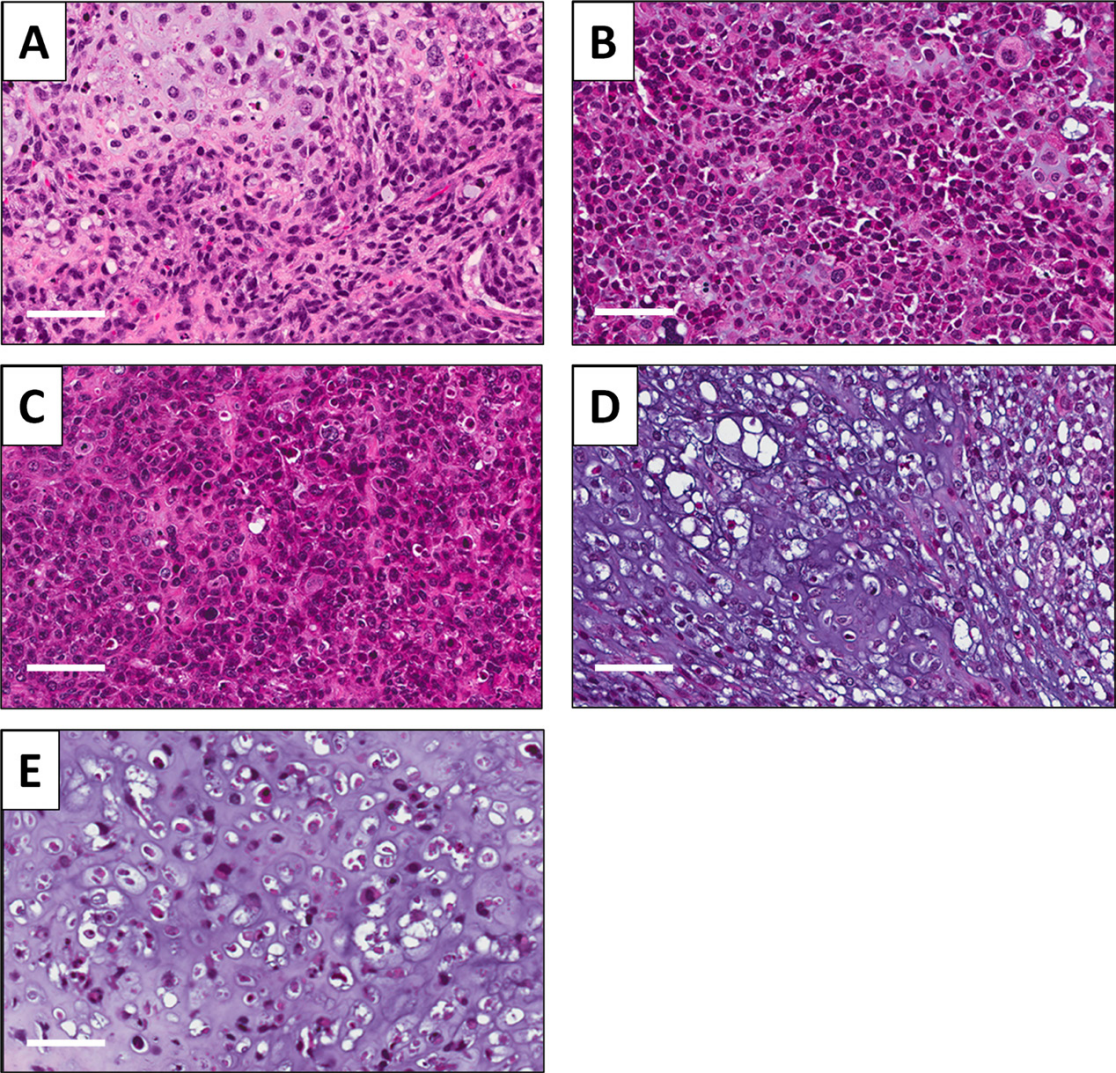
Effect of treatments on PDOX tumor histology Hematoxylin and eosin (H&E)-stained section of the (**A**) original patient tumor; (**B**) untreated PDOX tumor; (**C**) PDOX tumor treated with CDDP; (**D**) PDOX tumor treated with GEM+DOC; (**E**) PDOX tumor treated with TEM+IRN. Scale bars: 80 μm.

High power microscopy of the original patient tumor demonstrated neoplastic chondroid matrix occupied by anaplastic cells [8]. The tumor had hypercellular areas populated by anaplastic cells displaying nuclear pleomorphism, coarse and hyperchromatic chromatin and abundant mitotic figures (Figure 5A). High-power microscopy of the untreated PDOX tumor showed a solid and chondroblastic appearance similar to the patient's original tumor. Hypercellular areas were filled with cancer cells displaying nuclear pleomorphism and mitotic figures (Figure 5B) [8]. The PDOX tumor treated with CDDP comprised viable cells without apparent necrosis or inflammatory changes similar to the untreated control (Figure 5C). PDOX tumors treated with GEM+DOC showed changes in cancer-cell shape with slight areas of necrosis (Figure 5D). The TEM+IRN treated tumor showed more extensive tumor necrosis which is consistent with tumor regression after this treatment (Figure 5E).

In the largest soft-tissue sarcoma (STS) PDOX study to date, we previously demonstrated a 62% establishment rate among untreated high-grade sarcoma with a median establishment time of 54 days [40]. These results demonstrated that the PDOX model is a practical model for precision oncology for sarcoma patients.

The present results are a good example of precisely identifying a drug, the combination of TEM+ IRN that has potential for efficacy in the patient with CDDP-resistant lung metastatic osteosarcoma.

This result demonstrated the broad potential utility of the PDOX model for sarcoma and likely for other diseases as well with the goal of matching the patient with an effective drug or combination.

Previously-developed concepts and strategies of highly-selective tumor targeting can take advantage of molecular targeting of tumors, including tissue-selective therapy which focuses on unique differences between normal and tumor tissues [41–46].

## MATERIALS AND METHODS

### Mice

Athymic nu/nu nude mice (AntiCancer Inc., San Diego, CA), 4–6 weeks old, were used in this study. Animals were housed in a barrier facility on a high efficiency particulate arrestance (HEPA)-filtered rack under standard conditions of 12-hour light/dark cycles. The animals were fed an autoclaved laboratory rodent diet. All animal studies were conducted with an AntiCancer Institutional Animal Care and Use Committee (IACUC)-protocol specifically approved for this study and in accordance with the principles and procedures outlined in the National Institute of Health Guide for the Care and Use of Animals under Assurance Number A3873-1. In order to minimize any suffering of the animals, anesthesia and analgesics were used for all surgical experiments. Animals were anesthetized by subcutaneous injection of a 0.02 ml solution of 20 mg/kg ketamine, 15.2 mg/kg xylazine, and 0.48 mg/kg acepromazine maleate. The response of animals during surgery was monitored to ensure adequate depth of anesthesia. The animals were observed on a daily basis and humanely sacrificed by CO_2_ inhalation when they met the following humane endpoint criteria: severe tumor burden (more than 20 mm in diameter), prostration, significant body weight loss, difficulty breathing, rotational motion and body temperature drop [8].

### Patient-derived tumor

The study was previously reviewed and approved by the UCLA Institutional Review Board (IRB #10-001857). Written informed consent was previously obtained from the patient as part of the above-mentioned UCLA Institutional Review Board-approved protocol [8]. A 16-year old patient with localized left-distal-femoral high-grade osteosarcoma underwent CDDP-based neoadjuvant chemotherapy and limb-salvage distal-femoral replacement. The tumor necrosis extent of the primary tumor after CDDP based chemotherapy was 70%. One year later, the osteosarcoma relapsed with three bilateral metachronous pulmonary metastases. The patient was treated with curative surgery at the Division of Surgical Oncology, University of California, Los Angeles (UCLA). The patient did not receive neoadjuvant chemotherapy or radiotherapy prior to lung surgery [8].

### Surgical orthotopic implantation (SOI) for establishment of the PDOX osteosarcoma model

A lung metastasis from the osteosarcoma patient was previously established subcutaneously in mice [18]. Subcutaneously-grown tumors were harvested and cut into small fragments (3–4 mm). After nude mice were anesthetized, a 10 mm skin incision was made on the right thigh, the vastus lateralis muscle was opened and the biceps femoris muscle was split to reach the distal femur. An incision was made in the lateral patello-femoral ligament, sparing the knee joint and then the lateral condyle of the femur was resected. A single 3 to 4 mm tumor fragment was implanted orthotopically into the space to establish a PDOX model. The muscle and wound was closed with 6-0 nylon suture (Ethilon, Ethicon, Inc., NJ, USA) [8, 18].

### Treatment study design

The PDOX models were randomized into the following groups when tumor volume reached 100 mm^3^: G1, control without treatment; G2, CDDP (6 mg/kg, intraperitoneal (i.p.) injection, weekly, for 2 weeks); G3, GEM (100 mg/kg, i.p., weekly, for 2 weeks) combined with DOC (20 mg/kg, i.p., once); G4, TEM (25 mg/kg, p.o., daily, for 2 weeks) combined with IRN (4 mg/kg i.p., daily for 2 weeks) (Figure 1). Tumor sizes and body weight were measured with calipers and digital balance twice a week.

### Histological analysis

Fresh tumor samples were fixed in 10% formalin and embedded in paraffin before sectioning and staining. Tissue sections (3 μm) were deparaffinized in xylene and rehydrated in an ethanol series. Hematoxylin and eosin (H&E) staining was performed according to standard protocol. Histological examination was performed with a BHS system microscope. Images were acquired with INFINITY ANALYZE software (Lumenera Corporation, Ottawa, Canada) [8].

## References

[1] Bacci G, Ferrari S, Lari S, Mercuri M, Donati D, Longhi A, Forni C, Bertoni F, Versari M, Pignotti E (2002). Osteosarcoma of the limb: amputation or limb salvage in patients treated by neoadjuvant chemotherapy. J Bone Joint Surg Br.

[2] Muscolo DL, Ayerza MA, Aponte-Tinao LA, Ranalletta M (2004). Partial epiphyseal preservation and intercalary allograft reconstruction in high-grade metaphyseal osteosarcoma of the knee. J Bone Joint Surg Am.

[3] Simon MA, Aschliman MA, Thomas N, Mankin HJ (1986). Limb salvage treatment versus amputation for osteosarcoma of the distal end of the femur. J Bone Joint Surg Am.

[4] Lewis VO (2007). What's new in musculoskeletal oncology. J Bone Joint Surg Am.

[5] Meyers PA, Gorlick R, Heller G, Casper E, Lane J, Huvos AG, Healey JH (1998). Intensification of preoperative chemotherapy for osteogenic sarcoma: results of the Memorial Sloan-Kettering (T-12) protocol. J Clin Oncol.

[6] Fuchs N, Bielack SS, Epler D, Bieling P, Delling G, Körholz D, Graf N, Heise U, Jürgens H, Kotz R, Salzer-Kuntschik M, Weinel P, Werner M (1998). Long-term results of the cooperative German-Austrian-Swiss osteosarcoma study group's protocol COSS-86 of intensive multidrug chemotherapy and surgery for osteosarcoma of the limbs. Ann Oncol.

[7] Bacci G, Briccoli A, Ferrari S, Longhi A, Mercuri M, Capanna R, Donati D, Lari S, Forni C, DePaolis M (2001). Neoadjuvant chemotherapy for osteosarcoma of the extremity: long-term results of the Rizzoli's 4th protocol. Eur J Cancer.

[8] Igarashi K, Murakami T, Kawaguchi K, Kiyuna T, Miyake K, Zhang Y, Nelson SD, Dry SM, Li Y, Yanagawa J, Russell TA, Singh AS, Tsuchiya H (2017). A patient-derived orthotopic xenograft (PDOX) mouse model of an cisplatinum-resistant osteosarcoma lung metastasis that was sensitive to temozolomide and trabectedin: implications for precision oncology. Oncotarget.

[9] Engert F, Kovac M, Baumhoer D, Nathrath M, Fulda S (2017). Osteosarcoma cells with genetic signatures of BRCAness are susceptible to the PARP inhibitor talazoparib alone or in combination with chemotherapeutics. Oncotarget.

[10] Keir ST, Maris JM, Reynolds CP, Kang MH, Kolb EA, Gorlick R, Lock R, Carol H, Morton CL, Wu J, Kurmasheva RT, Houghton PJ, Smith MA (2013). Initial testing (stage 1) of temozolomide by the pediatric preclinical testing program. Pediatr Blood Cancer.

[11] Cuevas C, Francesch A (2009). Development of Yondelis (trabectedin, ET-743). A semisynthetic process solves the supply problem. Nat Prod Rep.

[12] Le Cesne A, Ray-Coquard I, Duffaud F, Chevreau C, Penel N, Bui Nguyen B, Piperno-Neumann S, Delcambre C, Rios M, Chaigneau L, Le Maignan C, Guillemet C, Bertucci F (2015). Trabectedin in patients with advanced soft tissue sarcoma: a retrospective national analysis of the French Sarcoma Group. Eur J Cancer.

[13] Demetri GD, von Mehren M, Jones RL, Hensley ML, Schuetze S, Staddon AP, Milhem MM, Elias AD, Ganjoo KN, Tawbi HA, Van Tine BA, Spira AI, Dean AP (2015). A randomized phase III study of trabectedin (T) or dacarbazine (D) for the treatment of patients (pts) with advanced liposarcoma (LPS) or leiomyosarcoma (LMS) [abstract]. J Clin Oncol.

[14] Germano G, Frapolli R, Belgiovine C, Anselmo A, Pesce S, Liguori M, Erba E, Uboldi S, Zucchetti M, Pasqualini F, Nebuloni M, van Rooijen N, Mortarini R (2013). Role of macrophage targeting in the antitumor activity of trabectedin. Cancer Cell.

[15] Scotlandi K, Perdichizzi S, Manara MC, Serra M, Benini S, Cerisano V, Strammiello R, Mercuri M, Reverter-Branchat G, Faircloth G, D'Incalci M, Picci P (2002). Effectiveness of Ecteinascidin-743 against drug-sensitive and -resistant bone tumor cells. Clin Cancer Res.

[16] https://www.cancer.gov/news-events/cancer-currents-blog/2015/fda-trabectedin-sarcoma.

[17] Laverdiere C, Kolb EA, Supko JG, Gorlick R, Meyers PA, Maki RG, Wexler L, Demetri GD, Healey JH, Huvos AG, Goorin AM, Bagatell R, Ruiz-Casado A (2003). Phase II study of ecteinascidin 743 in heavily pretreated patients with recurrent osteosarcoma. Cancer.

[18] Murakami T, Igarashi K, Kawaguchi K, Kiyuna T, Zhang Y, Zhao M, Hiroshima Y, Nelson SD, Dry SM, Li Y, Yanagawa J, Russell T, Federman N (2017). Tumor-targeting Salmonella typhimurium A1-R regresses an osteosarcoma in a patient-derived xenograft model resistant to a molecular-targeting drug. Oncotarget.

[19] Hiroshima Y, Zhang Y, Murakami T, Maawy A, Miwa S, Yamamoto M, Yano S, Sato S, Momiyama M, Mori R, Matsuyama R, Chishima T, Tanaka K (2014). Efficacy of tumor-targeting Salmonella typhimurium A1-R in combination with anti-angiogenesis therapy on a pancreatic cancer patient-derived orthotopic xenograph (PDOX) and cell line mouse models. Oncotarget.

[20] Fu X, Guadagni F, Hoffman RM (1992). A metastatic nude-mouse model of human pancreatic cancer constructed orthotopically with histologically intact patient specimens. Proc Natl Acad Sci U S A.

[21] Hiroshima Y, Maawy A, Zhang Y, Murakami T, Momiyama M, Mori R, Matsuyama R, Katz MH, Fleming JB, Chishima T, Tanaka K, Ichikawa Y, Endo I (2014). Metastatic recurrence in a pancreatic cancer patient derived orthotopic xenograft (PDOX) nude mouse model is inhibited by neoadjuvant chemotherapy in combination with fluorescence-guided surgery with an anti-CA 19-9-conjugated fluorophore. PLoS One.

[22] Hiroshima Y, Maawy AA, Katz MH, Fleming JB, Bouvet M, Endo I, Hoffman RM (2015). Selective efficacy of zoledronic acid on metastasis in a patient-derived orthotopic xenograph (PDOX) nude-mouse model of human pancreatic cancer. J Surg Oncol.

[23] Fu X, Le P, Hoffma RM (1993). A metastatic-orthotopic transplant nude-mouse model of human patient breast cancer. Anticancer Res.

[24] Fu X, Hoffman RM (1993). Human ovarian carcinoma metastatic models constructed in nude mice by orthotopic transplantation of histologically-intact patient specimens. Anticancer Res.

[25] Wang X, Fu X, Hoffman RM (1992). A new patient-like metastatic model of human lung cancer constructed orthotopically with intact tissue via thoracotomy in immunodeficient mice. Int J Cancer.

[26] Hiroshima Y, Zhang Y, Zhang N, Maawy A, Mii S, Yamamoto M, Uehara F, Miwa S, Yano S, Murakami T, Momiyama M, Chishima T, Tanaka K (2015). Establishment of a patient-derived orthotopic xenograph (PDOX) model of HER-2-positive cervical cancer expressing the clinical metastatic pattern. PLoS One.

[27] Fu XY, Besterman JM, Monosov A, Hoffman RM (1991). Models of human metastatic colon cancer in nude mice orthotopically constructed by using histologically intact patient specimens. Proc Natl Acad Sci U S A.

[28] Metildi CA, Kaushal S, Luiken GA, Talamini MA, Hoffman RM, Bouvet M (2014). Fluorescently-labeled chimeric anti-CEA antibody improves detection and resection of human colon cancer in a patient-derived orthotopic xenograft (PDOX) nude mouse model. J Surg Oncol.

[29] Hiroshima Y, Maawy A, Metildi CA, Zhang Y, Uehara F, Miwa S, Yano S, Sato S, Murakami T, Momiyama M, Chishima T, Tanaka K, Bouvet M (2014). Successful fluorescence-guided surgery on human colon cancer patient-derived orthotopic xenograft mouse models using a fluorophore-conjugated anti-CEA antibody and a portable imaging system. J Laparoendosc Adv Surg Tech A.

[30] Furukawa T, Kubota T, Watanabe M, Kitajima M, Hoffman RM (1993). Orthotopic transplantation of histologically intact clinical specimens of stomach cancer to nude mice: correlation of metastatic sites in mouse and individual patient donors. Int J Cancer.

[31] Murakami T, DeLong J, Eilber FC, Zhao M, Zhang Y, Zhang N, Singh A, Russell T, Deng S, Reynoso J, Quan C, Hiroshima Y, Matsuyama R (2016). Tumor-targeting Salmonella typhimurium A1-R in combination with doxorubicin eradicate soft tissue sarcoma in a patient-derived orthotopic xenograft PDOX model. Oncotarget.

[32] Hiroshima Y, Zhao M, Zhang Y, Zhang N, Maawy A, Murakami T, Mii S, Uehara F, Yamamoto M, Miwa S, Yano S, Momiyama M, Mori R (2015). Tumor-targeting Salmonella typhimurium A1-R arrests a chemo-resistant patient soft-tissue sarcoma in nude mice. PLoS One.

[33] Kiyuna T, Murakami T, Tome Y, Kawaguchi K, Igarashi K, Zhang Y, Zhao M, Li Y, Bouvet M, Kanaya F, Singh A, Dry S, Eilber FC (2016). High efficacy of tumor-targeting Salmonella typhimurium A1-R on a doxorubicin- and dactolisib-resistant follicular dendritic-cell sarcoma in a patient-derived orthotopic xenograft PDOX nude mouse model. Oncotarget.

[34] Murakami T, Singh AS, Kiyuna T, Dry SM, Li Y, James AW, Igarashi K, Kawaguchi K, DeLong JC, Zhang Y, Hiroshima Y, Russell T, Eckardt MA (2016). Effective molecular targeting of CDK4/6 and IGF-1R in a rare FUS-ERG fusion CDKN2A-deletion doxorubicin-resistant Ewing's sarcoma in a patient-derived orthotopic xenograft (PDOX) nude-mouse model. Oncotarget.

[35] Hiroshima Y, Zhang Y, Zhang N, Uehara F, Maawy A, Murakami T, Mii S, Yamamoto M, Miwa S, Yano S, Momiyama M, Mori R, Matsuyama R (2015). Patient-derived orthotopic xenograft (PDOX) nude mouse model of soft-tissue sarcoma more closely mimics the patient behavior in contrast to the subcutaneous ectopic model. Anticancer Res.

[36] Yamamoto M, Zhao M, Hiroshima Y, Zhang Y, Shurell E, Eilber FC, Bouvet M, Noda M, Hoffman RM (2016). Efficacy of tumor-targeting Salmonella typhimurium A1-R on a melanoma patient-derived orthotopic xenograft (PDOX) nude-mouse model. PLoS One.

[37] Kawaguchi K, Murakami T, Chmielowski B, Igarashi K, Kiyuna T, Unno M, Nelson SD, Russell TA, Dry SM, Li Y, Eilber FC, Hoffman RM (2016). Vemurafenib-resistant BRAF-V600E mutated melanoma is regressed by MEK targeting drug trametinib, but not cobimetinib in a patient-derived orthotopic xenograft (PDOX) mouse model. Oncotarget.

[38] Kawaguchi K, Igarashi K, Murakami T, Chmiewloski B, Kiyuna T, Zhao M, Zhang Y, Singh A, Unno M, Nelson SD, Russell T, Dry SM, Li Y (2016). Tumor-targeting Salmonella typhimurium A1-R combined with Temozolomide regresses malignant melanoma with a BRAF-V600 mutation in a patient-derived orthotopic xenograft (PDOX) model. Oncotarget.

[39] Kurmasheva RT, Houghton PJ, Hoffman R.M., Coleman W.B., Tsongalis G.J. (2017). Chapter 11: The use of pediatric patient-derived xenografts for identifying novel agents and combination.. Patient-Derived Mouse Models of Cancer.

[40] Russell TA, Eckardt M, Murakami T, Elliott I, Kawaguchi K, Kiyuna T, Igarashi K, Li Y, Crompton J, Graham DS, Dry SM, Bernthal N, Yanagawa J Clinical factors impacting the establishment of soft tissue sarcoma patient-derived orthotopic xenograft (PDOX): A UCLA sarcoma program prospective clinical trial. JCO Precision Oncology.

[41] Blagosklonny MV (2003). Matching targets for selective cancer therapy. Drug Discov Today.

[42] Blagosklonny MV (2005). Teratogens as anti-cancer drugs. Cell Cycle.

[43] Blagosklonny MV (2001). Treatment with inhibitors of caspases, that are substrates of drug transporters, selectively permits chemotherapy-induced apoptosis in multidrug-resistant cells but protects normal cells. Leukemia.

[44] Blagosklonny MV (2006). Target for cancer therapy: proliferating cells or stem cells. Leukemia.

[45] Apontes P, Leontieva OV, Demidenko ZN, Li F, Blagosklonny MV (2011). Exploring long-term protection of normal human fibroblasts and epithelial cells from chemotherapy in cell culture. Oncotarget.

[46] Blagosklonny MV (2003). Tissue-selective therapy of cancer. Br J Cancer.

